# Plasma thymus and activation‐regulated chemokine (TARC) as diagnostic marker in pediatric Hodgkin lymphoma

**DOI:** 10.1002/jha2.41

**Published:** 2020-07-04

**Authors:** Eline A. M. Zijtregtop, Friederike Meyer‐Wentrup, Wai‐Chu Wong, Raoull Hoogendijk, Marta Lopez‐Yurda, Christian M. Zwaan, Auke Beishuizen

**Affiliations:** ^1^ Department of Pediatric Hematology and Oncology Erasmus Medical Centre ‐ Sophia Children's Hospital Rotterdam The Netherlands; ^2^ Department of Hemato‐oncology Princess Máxima Centre for Pediatric Oncology Utrecht The Netherlands; ^3^ Department of Biometrics Netherlands Cancer Institute Amsterdam The Netherlands

## Abstract

Pediatric classical Hodgkin's lymphoma (cHL) is characterized by Hodgkin Reed‐Sternberg cells located in an inflammatory microenvironment. Blood biomarkers result from active crosstalk between these cells. One promising biomarker in adult cHL patients is “thymus‐and‐activation‐regulated chemokine” (TARC). The objectives of this study were to define normal TARC values in non‐cHL children and to investigate and correlate pretherapy TARC as diagnostic marker in pediatric cHL. In this multicenter prospective study, plasma and serum samples were collected of newly diagnosed cHL patients before start of treatment (n = 49), and from randomly selected non‐cHL patients (n = 81). TARC levels were measured by enzyme‐linked immunosorbent assay. The non‐cHL patients had a median plasma TARC value of 71 pg/mL (range: 18‐762), compared to 14 619 pg/mL (range: 380‐73 174) in cHL patients (*P* < .001). TARC values had a high discriminatory power (AUC = .999; 95% confidence interval, .998‐1). A TARC cutoff level of 942 pg/mL maximized the sum of sensitivity (97.9%) and specificity (100%). TARC plasma levels were associated with age, treatment level, bulky disease, B‐symptoms, and erythrocyte sedimentation rate. TARC was found to be a highly specific and sensitive diagnostic marker for pediatric cHL. This noninvasive marker could be of great value as screening test in the work‐up for pediatric patients with lymphadenopathy.

## 1 INTRODUCTION

Classical Hodgkin lymphoma (cHL) is a clonal postgerminal center B‐cell malignancy and a common lymphoma in children and adolescents. Malignant Hodgkin and Reed‐Sternberg (HRS) cells account for only 0.1‐10% of all cells present in tumor tissue. They are by far outnumbered by reactive immune cells, such as activated T‐cells, histiocytes, macrophages, fibroblasts, and eosinophils [1, 2, 3]. This microenvironment is of major importance for growth, proliferation, and survival of HRS cells. HRS cells and microenvironment communicate through expression of chemo‐ and cytokines, which can be detected in blood and may function as surrogate markers for lymphoma viability [1, 4].

The gold standard for diagnosing HL is by lymph node biopsy. This is a relatively time‐consuming and expensive procedure, requiring general anesthesia that in patients with mediastinal mass may be contraindicated due to danger of causing respiratory failure. After the diagnosis has been made, staging in pediatric cHL patients requires Fluor‐Deoxyglucose‐Positron Emission Tomography (FDG‐PET) scans. Although the latter is a sensitive test, it carries several disadvantages, including exposure to radiation, time consumption, high costs, and lack of specificity [5]. Blood biomarkers on the contrary are more easily available, cost‐effective, and almost noninvasive for the patient. Therefore, they could be of great value to diagnose and stage pediatric cHL.

One biomarker reported in adult patients with cHL is thymus and activation‐regulated chemokine (TARC; also termed CCL17) [6, 7, 8]. TARC is produced by HRS cells and antigen‐presenting cells and attracts T‐helper type 2 cells [9]. In adults, approximately 90% of cHL patients show positive TARC staining of HRS cells by immunohistochemistry and about 82‐93% of patients have significantly elevated TARC levels in their pretreatment serum [10, 11, 12, 13]. TARC is not expressed in adult patients with nonclassical nodular lymphocyte predominant Hodgkin lymphomas or non‐Hodgkin lymphomas [14, 15]. In adult cHL patients, higher TARC levels are correlated with presence of B‐symptoms, bulky disease, metabolic tumor volume, and advanced disease stage [13]. Moreover, TARC levels decrease after one cycle of chemotherapy in both early and advanced stage cHL patients [6, 13].

To our knowledge, TARC levels in pediatric patients with cHL have not been reported. Besides, normal values of TARC are not yet determined in children and may differ from those in adults. Because pediatric and adult cHL differ partly with respect to histology, risk factors, clinical presentation, and staging at diagnosis and prognosis [16, 17, 18], further research is warranted to determine whether TARC is a diagnostic biomarker in pediatric cHL patients as well. Therefore, the aim of this study is to determine normal TARC values in children without cHL (non‐cHL) and to investigate whether TARC can be used as a diagnostic marker in pediatric cHL patients, using biopsy results as gold standard. Moreover, we study the association of TARC values with stage of disease, presence of B symptoms, erythrocyte sedimentation rate (ESR), C‐reactive protein (CRP), and bulky disease.

## 2 METHODS

### 2.1 Patient inclusion

This multicenter prospective study was sponsored by and conducted in the Erasmus Medical Centre—Sophia Children's Hospital (EMC—Sophia) in Rotterdam, in the Princess Máxima Centre for Pediatric Oncology in Utrecht, and in the Amsterdam University Medical Centre, location VUmc, in Amsterdam (all in the Netherlands). Serial plasma and serum samples were collected prospectively from newly diagnosed pediatric cHL patients from January 2016 till March 2020. Samples for the non‐cHL group were collected from October 2017 until August 2019.

Inclusion criteria for both newly diagnosed and relapsed cHL patients were as follows: (a) diagnosis of cHL confirmed by reference pathology, (b) aged below 18 years, (c) receiving standard treatment according to the European Network of Pediatric Hodgkin's Lymphoma First (interim phase) and Second International Inter‐Group study for Classical Hodgkin's Lymphoma in Children and Adolescents (EuroNet‐PHL C1(interim) or C2, EudraCT number 2006‐000995‐33 and 2012‐004053‐88) protocol or treatment for relapsed or refractory patients, (d) availability of a blood (plasma and/or serum) sample before start of treatment, and (e) written informed consent of the patient and/or the patient's parents or guardians according to national laws. Exclusion criteria were HIV positivity or underlying immunologic disorder except for an (medical history of) Epstein Barr Virus infection. The diagnosis of cHL was made by lymph node biopsy, confirmed both by a local pathologist and by Dutch central review. All patients were staged and evaluated to conform the Cotswold revision of the Ann Arbor staging criteria with FDG‐PET and MRI/CT imaging [19]. Patients were stratified into three different treatment levels based on staging and the following risk factors: ESR level above 30 mm/h, the presence of B‐symptoms, bulky disease, and E‐lesions. B‐symptoms were unexplained fever, weight loss (>10% in 6 months), or drenching night sweats. The definition of bulk was the volume of the largest contiguous lymph node mass as ≥200 mL. An E‐lesion is a contiguous infiltration of a lymph node mass into extra‐lymphatic structures or organs (eg, lung and bone). Baseline characteristics including age, sex, presence of B‐symptoms, ESR, CRP, stage, bulky disease, and E‐lesions were collected.

The non‐cHL group consisted of randomly selected patients from different departments, including hematological, oncological, and endocrinological outpatient clinics, excluding children with cHL. This non‐cHL group was treated in EMC‐Sophia. Inclusion criteria were (a) age below 18 years, (b) no diagnosis of cHL, (c) written informed consent of the patient and/or the patient's parents or guardians according to national laws, and (d) with a scheduled blood draw. Exclusion criteria were HIV positivity or underlying immunologic disorder except for an (medical history of) Epstein Barr Virus infection. Atopic dermatitis and eczema can cause elevated TARC levels in both children and adults. Therefore, these diseases were checked and recorded [20, 21, 22].

This study was IRB‐approved, and registered under Dutch Trial registry number 6876.

### 2.2 Plasma and serum collection

Recently, Zhao et al showed that TARC levels are significantly lower in platelet‐free plasma samples than in platelet containing serum samples, because platelets contain TARC. [23, 24]. To investigate whether plasma and serum are both a useful source of TARC and to compare their reliability as diagnostic markers, we collected serum and plasma samples for each participant in the study.

### 2.3 TARC detection

After collection, serum tubes were stored at room temperature in a vertical position prior to centrifugation. Plasma tubes were centrifuged within 30 min after arrival at the laboratory. Blood sample centrifugation was performed at room temperature for 15 min at 1000 × *g*. Afterward serum and plasma supernatant was collected and frozen immediately at –80°C. TARC levels were measured by enzyme‐linked immunosorbent assay (ELISA) (R&D systems, USA, Human CCL 17/TARC Quantikine ELISA Kit).

TARC measurement by ELISA technology was performed in the laboratory of Pediatric Oncology at the EMC—Sophia, Rotterdam. Since June 2018, all measurements were performed at the diagnostic laboratory of the Princess Máxima Centre, and the Laboratory of Translational Immunology of the Wilhelmina Children's Hospital in Utrecht. For quality assessment, 20 samples were tested in both laboratories to make sure TARC levels were comparable.

### 2.4 Statistical analyses

Statistical comparison of categorical variables was done using Pearson's chi‐square test for differences in the distribution of categorical variables (Fisher's exact test in case of sparse data), and the Mann‐Whitney *U*‐test for differences in continuous variables. For differences in continuous variables in paired samples (TARC plasma vs TARC serum), Wilcoxon's signed‐rank test was used.

Simple and multiple linear regression was used to investigate associations between clinical characteristics and (log) TARC levels. Residual diagnostics were used to assess adequacy of model fit.

To evaluate how TARC levels discriminate cHL patients from the non‐cHL controls, a receiver operating characteristic (ROC) curve analysis was performed. For age‐matched cHL patients and non‐cHL controls, a covariate‐adjusted ROC curve was estimated. Diagnostic accuracy was given by the area under the ROC curve (AUC), and leave‐one‐out cross validation (LOOCV) was implemented. The optimal threshold was determined based on Youden's index, which maximizes the sum of sensitivity and specificity. Before study start, it was obtained that with at least 40 cHL patients available, a 90% two‐sided confidence interval for sensitivity with lower limit 85% would be produced when the sample sensitivity is 95% (analogously for non‐cHL controls and specificity).

To compare TARC plasma and serum AUCs, DeLong's method for paired data AUCs was used.

All statistical tests were two‐sided. We used R (version 3.4.1) and SAS (version 9.4) for analyses.

## 3 RESULTS

### 3.1 Patient's characteristics

For the non‐cHL group, plasma and serum samples were collected from 82 children. Two patients had to be excluded, one because the samples were hemolytic and one because no samples were available. For the cHL patient group, 53 patients were included (52 with primary diagnosis of cHL, one with relapsed cHL). Six cHL patients had to be excluded because there were no pretreatment samples available. Plasma TARC data were available for 47 patients. For serum analysis, 13 patients had to be excluded due to absence of pretreatment samples. Serum TARC data were available for 40 patients.

Baseline characteristics of the 47 newly diagnosed cHL patients, including a comparison with patient data from the GPOH‐HD‐2002 study, used as a representative sample of cHL patients, are summarized in Table 1. Patients excluded from the analysis were compared with included patients. There were no significant differences in baseline characteristics between these groups. Moreover, comparison of baseline characteristics of the study group with the reference study cohort of 573 children with cHL in the GPOH‐HD‐2002 study showed no significant differences in age, sex, and the presence of B‐symptoms. However, our study population had a significantly different distribution of stages with less patients in stage I and II, and more patients in stage III and IV (*P* = .012).

**TABLE 1:** Baseline characteristics cHL group

	Plasma TARC (N = 47)	Serum TARC (N = 40)	GPOH‐HD‐2002 study (N = 573)	*P*‐value
Age in years median (range)	15 (6‐17)	15 (6‐17)		
< 13 years (%)	11 (23.4)	10 (25.0)	169 (29.5)	.473
≥ 13 years (%)	36 (76.6)	30 (75.0)	404 (70.5)	
Male (%)	20 (42.6)	20 (48.8)	287 (50.1)	.4
Atopic dermatitis/eczema (%)^a^	4 (8.5)	4 (8.8)		
B‐symptoms^a^	22 (47.8)	18 (45.0)	218 (38.0)	.249
Fever (%)	11 (23.9)	10 (25.0)		
Drenching night sweats (%)	16 (34.8)	11 (30.0)		
Weight loss (%)	9 (19.6)	9 (22.5)		
ESR > 30 mm/h^b^	30 (65.2)	23 (57.5)		
Stage^a^				
1 (%)	0 (0.0)	0 (0.0)	14 (2.4)	
2 (%)	15 (32.6)	12 (30.0)	313 (54.6)	.012
3 (%)	15 (32.6)	13 (32.5)	110 (19.2)	
4 (%)	16 (34.8)	15 (37.5)	126 (23.7)	
E‐lesions (%)^c^	5 (11.1)	4 (10.3)		
Bulky disease (%)^a^	17 (37.0)	14 (35.0)		
Treatment level^a^				
1 (%)	6 (13.0)	6 (15.0)		
2 (%)	12 (26.1)	10 (25.0)		
3 (%)	27 (58.7)	23 (57.5)		
Other	1 (2.2)	1 (2.5)		

a Data missing for one patient.

b Not performed in our hospital in three patients.

c Data missing for two patients.

The median age of the non‐cHL group was 13 (range: 1‐17) years. Slightly less male patients (39; 48, 8%) were included. Atopic dermatitis was found in eight (10%) patients.

### 3.2 Normal TARC values in non‐cHL group

Non‐cHL patients had a median TARC value of 71 (range: 18‐762) pg/mL for plasma and 317 (range: 27‐1300) pg/mL for serum. TARC levels were associated with age: for 1‐year increase in age, a 4% decrease in TARC plasma was found, and a 4% decrease for TARC serum. We did not detect elevated TARC plasma and serum levels in the eight cases of atopic dermatitis in this group (Table S1).

### 3.3 TARC levels are highly elevated in the blood of pediatric cHL patients

The median pretreatment plasma TARC level in 47 cHL patients was 14 619 pg/mL (range: 380‐73 174). All but one patient had elevated TARC levels (97.8%).

The median serum pretreatment TARC level in 40 cHL patients was 38 263.5 pg/mL (range: 2257‐176 451). Thirty‐nine patients had elevated TARC levels (97.2%).

### 3.4 Elevated TARC levels are a specific and sensitive predictor of cHL in children

**FIGURE 1:**
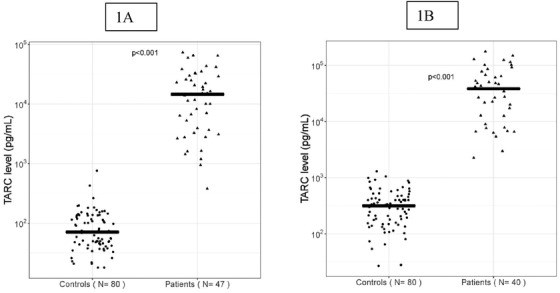
Plasma and serum TARC levels are significantly higher in cHL patients than in non‐cHL patients. TARC expression in plasma and serum from newly diagnosed cHL patients and non‐cHL patients. A, Plasma TARC levels among 80 non‐cHL patients and 47 pretreatment newly diagnosed cHL patients. The median plasma TARC level was 71 pg/mL (range: 18‐762) and 14 619 pg/mL (range: 380‐73 174), respectively, in the non‐cHL and the cHL patients. Pretreatment patient samples were significantly higher compared to the non‐cHL patients (*P* < .001). B, Serum TARC levels among 80 non‐cHL patients and 40 pretreatment newly diagnosed cHL patients. The median serum TARC level was 317 pg/mL (range: 27‐1300) and 38 263 pg/mL (range: 2257‐176 451) in the non‐cHL and the cHL patients. Pretreatment patient samples were significantly higher compared to the non‐cHL patients (*P* < .001)

**FIGURE 2:**
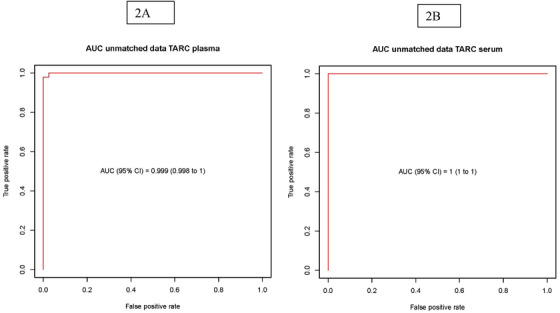
Serum and plasma TARC levels are sensitive and specific biomarkers in cHL patients, both unmatched and matched data. Receiver operating characteristics (ROC) analysis of plasma and serum TARC levels. A, A TARC cutoff level of 942 pg/mL maximized the sum of sensitivity (97.9%; 95% confidence interval [CI], 88.7‐100%) and specificity (100%; 95% CI, 95.5‐100%) (AUC = .999; 95% CI, .998‐1). B, For serum, with a cutoff level of 2257/mL, the sum of sensitivity (100%; 95% CI, 91.2‐100%) and specificity (100%; 95% CI, 95.5‐100%) was maximized (AUC = 1)

TARC plasma and serum levels in cHL patients were significantly higher (*P* < .001) than in non‐cHL patients (Figure 1). TARC plasma values had a high discriminatory power: AUC = .999; 95% CI, .998‐1 and LOOCV AUC = .999; 95% CI, .997‐1 (Figure 2A). A TARC cutoff level of 942 pg/mL maximized the sum of sensitivity (97.9%; 95% CI, 88.7‐100%) and specificity (100%; 95% CI, 95.5‐100%). The AUC remained high (>0.99) when calculated per treatment level (for serum, AUC = 1; Figure 2B). For a cutoff level of 2257 pg/mL, the sum of sensitivity (100%; 95% CI. 91.2‐100%) and specificity (100%; 95% CI, 95.5‐100%) was maximized.

The non‐cHL group had a higher representativeness of younger age strata than the cHL group. Median age was 12 (range: 0‐18) in non‐cHL patients compared to 15 (range: 7‐18) in cHL patients (*P* = .002). In order to improve statistical precision, analyses 1:1 matching cHL to non‐cHL patients by age were also performed, although this lead to a reduction of the number of cHL patients used in the plasma analyses from 47 to 39. For the matched dataset, AUC = .999 (95% CI, .998‐1) and LOOCV AUC = .996 (95% CI, .989‐1) for plasma. The age‐dependent cutoffs ranged from 742.2 to 1108.5 (for serum, analyses were based on 32 cHL patients, with AUC = .997 [95% CI, .991‐1] and LOOCV AUC = .993 [95% CI, .979‐1]). Cutoffs ranged from 3300.13 to 4100.28.

No difference was found in the AUCs for the correlated ROC curves of paired plasma and serum data (*P* = .413).

### 3.5 TARC levels correlate with parameters of tumor burden

We assessed the association between TARC levels and disease characteristics (Figure 3 and Tables S2 and S3). Patients with bulky disease had significantly higher plasma and serum TARC levels compared to patients without (approximately threefold increase for plasma and serum). Plasma and serum TARC levels were significantly associated with the presence of B‐symptoms at diagnosis (91% and 40% increase for plasma and serum, respectively) and ESR > 30 mm/h (approximately three‐ and twofold increase for plasma and serum, respectively). Patients with a higher treatment level and patients with organ involvement and E‐lesions had significantly higher plasma but not serum TARC levels (slightly less than twofold increase or higher).

FIGURE 3TARC levels are correlated with disease burden. A, Patients with bulky disease had significantly higher plasma and serum TARC levels compared to patients without bulky disease (215% and 192% increase for plasma and serum, respectively). B, Plasma and serum TARC levels were significantly associated with the presence of B‐symptoms at diagnosis (91% and 40% increase for plasma and serum, respectively). C, Plasma and serum TARC levels were associated with ESR > 30 mm/h (215% and 95% increase for plasma and serum, respectively). D, Patients with a higher treatment level and patients with organ involvement and E‐lesions had significantly higher plasma TARC levels (at least 190% increase), but this was not the case for serum TARC
^*^Other: The patient with relapsed HL was treated with a relapsed treatment protocol where treatment levels were not used.

**Figure:**
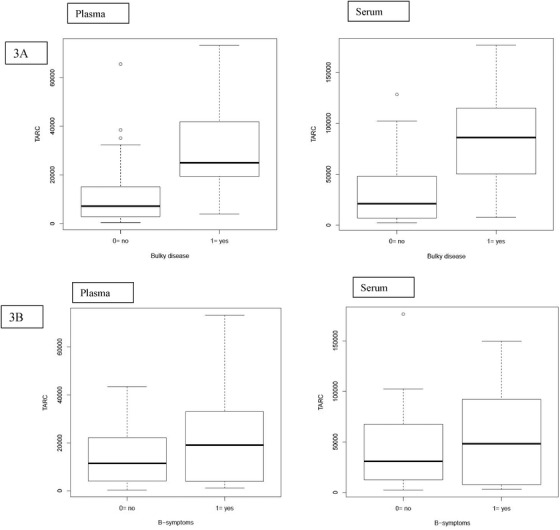

**Figure:**
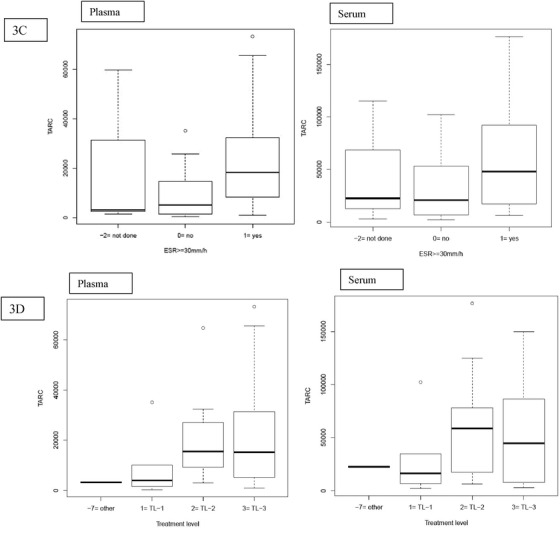

No significant associations were found with CRP or stage. The fact that TARC is associated with treatment level but not with stage could be due to the strong correlation of TARC with bulky disease, B‐symptoms, and ESR, characteristics that are used to determine treatment level. When introducing these three variables together with stage in a multiple regression model, bulky disease remains significantly associated with TARC plasma and serum levels (slightly less than threefold increase or higher in TARC for presence of bulky disease vs not).

## 4 DISCUSSION

This is the first study to report normal TARC values in children. We define a cutoff level for TARC elevation in children with cHL. Our data demonstrate that TARC level in plasma or serum is a sensitive and specific diagnostic marker for pediatric cHL, with histology as gold standard. These results are even more promising than the results of TARC levels in adult patients with cHL [6, 7, 8]. One patient with low stage of disease (II) did not show elevated TARC in both plasma and serum. This patient had no B‐symptoms, no elevated ESR, no E‐lesions, and no bulky disease. We hypothesize that serum and plasma TARC levels in that child could not be detected due to scarcity of HRS cells and low tumor volume.

Because TARC is produced by HRS cells, we hypothesized that plasma TARC levels would correlate with tumor burden (defined by systemic symptoms and amount of tumor tissue) and staging. Consistent with adult studies, TARC levels in pediatric cHL patients correlated with bulky disease [10, 11, 13] at diagnosis, and with B‐symptoms and ESR [11, 13, 25]. A correlation with disease stage was not found unlike results from most studies in adults [11, 13, 26]. We detect a trend toward higher levels of TARC in stage 3 and 4 disease, compared to stage 2 disease, although this is not significant. There were no patients with stage 1 disease and low patient numbers with stage 2 disease included in this study. This may explain why we did not find a correlation of TARC levels with disease stage. However, we did find a correlation between TARC and treatment levels according to the EuroNet‐PHL‐C2 protocol, which is a derivative of staging that takes into account systemic signs of inflammation such as B‐symptoms, ESR level, bulky disease, and presence of E‐lesions. The fact that TARC is associated with treatment level but not with stage could be due to the strong correlation of TARC with bulky disease.

TARC levels in pediatric non‐cHL patients closely correspond to TARC levels of adult non‐cHL patients investigated in other studies [11, 23]. TARC levels found in the non‐cHL group are very consistent and stay in a comparable range. We therefore think that these patients form a representative non‐cHL group. In our non‐cHL group, TARC plasma and serum levels decreased with age; further research is necessary to explain this.

In contrast to earlier studies, where children and adult patients with atopic dermatitis and eczema had elevated levels of TARC [20, 21, 22, 23]; this was not the case in the eight non‐cHL patients with atopic dermatitis included in our study.

TARC is measurable in both plasma and serum, although normal values and cutoff levels are different. No difference was found in the AUCs for the correlated ROC curves of paired plasma and serum data. Therefore, both serum and plasma can be used to measure TARC as diagnostic marker.

In this study, plasma and serum samples were all taken before start of treatment with chemotherapy. A possible limitation of this study is the fact that in the majority of cases, a complete lymph node was removed for pathological diagnosis before the TARC plasma and serum samples were drawn. In patients with lower tumor burden and stage, this might have influenced the amount of TARC, because TARC is produced by HRS cells present in the lymph nodes.

We propose that blood TARC levels can be of great value as screening test in the diagnostic work‐up for pediatric patients with lymphadenopathy. Especially cHL is sometimes difficult to differentiate from other pathologies due to the slow growing pattern and, sometimes, the lack of specific symptoms. Diagnostic markers such as ESR are useful but nonspecific in this work‐up. Based on this study, elevated TARC has high sensitivity and specificity for cHL. Further research in patients with other causes of lymphadenopathy is necessary to define how TARC should be implemented in the diagnostic algorithm.

In conclusion, our study shows that TARC is a valuable diagnostic biomarker for children with cHL, with a sensitivity of 97.9% and specificity of 100% for plasma (cutoff level of 942 pg/mL) and 100% sensitivity and 100% specificity for serum (cutoff level of 2257 pg/mL). TARC levels are correlated with bulky disease, treatment level, the presence of B‐symptoms, and ESR.

## AUTHOR CONTRIBUTIONS

AB and FM‐W conceived the project and provided leadership. EZ, RH, WW, FM‐W, and AB organized the study. WW collected the non‐cHL group samples. EZ, RH, and ML analyzed the data and contributed to the manuscript. ML made the figures. EZ wrote the manuscript. FM‐W and AB supervised the study. All authors reviewed and accepted the contents of the article.

## CONFLICT OF INTEREST

The authors declare no conflict of interest.

## DATA SHARING

Please contact the corresponding author for access to original data.

## Supporting information

Supporting Information
