# Supplementary material for: Plasma thymus and activation‐regulated chemokine (TARC) as diagnostic marker in pediatric Hodgkin lymphoma
Source: EJHaem. 2020 Jul 4;1(1):152–60. doi: 10.1002/jha2.41 (PMC9176129; doi:10.1002/jha2.41)
Supplement: Supplementary file 1 — Supporting Information [file JHA2-1-152-s001.docx]

**Supplement table 1. Regression results for TARC plasma and disease burden (non-cHL group only)**

|  | **Dependent variable: log(TARC)** | | | |
| --- | --- | --- | --- | --- |
|  | Plasma, simple regression | % TARC plasma increase (decrease if negative) | Serum, simple regression | % TARC serum increase (decrease if negative) |
| Age (years) | -0.036†  (-0.064, -0.008) | -4 | -0.042†  (-0.075, -0.008) | -4 |
| Age  >=13 years (ref. <13 years) | -0.467‡  (-0.766, -0.168) | -37 | -0.598‡  (-0.944, -0.253) | -45 |
| Eczema  1= yes (ref. 0= no) | 0.029  (-0.493, 0.551) | 3 | 0.310  (-0.296, 0.917) | 36 |

* p<0.1

† p<0.05

‡ p< 0.01

**Supplement table 2. Regression results for TARC plasma and disease burden (cHL patients only)**

|  | **Dependent variable: log(TARC plasma)** | | | |
| --- | --- | --- | --- | --- |
|  | Simple regression | Multiple regression | % TARC increase (simple) | % TARC increase (multiple) |
| Age (years) | 0.244‡  (0.125, 0.362) |  | 28 |  |
| Age  >=13 years  (ref. <13 years) | 1.565‡  (0.811, 2.319) |  | 378 |  |
| Stage 3= III  (ref. 2= II) | 0.314  (-0.627, 1.256) | 0.385  (-0.420, 1.190) | 37 |  |
| Stage 4= IV  (ref. 2= II) | 0.082  (-0.859, 1.024) | -0.349  (-1.173, 0.474) | 9 | -29 |
| CRP | 0.006  (-0.003, 0.016) |  | 1 |  |
| ESR | 0.014‡  (0.004, 0.023) |  | 1 |  |
| ESR 1= yes  (ref. 0= no) | 1.147‡  (0.402, 1.893) | 0.646  (-0.209, 1.500) | 215 | 91 |
| Bulky disease 1= yes  (ref. 0= no) | 1.147‡  (0.434, 1.860) | 1.045†  (0.285, 1.806) | 215 | 184 |
| B-symptoms 1= yes  (ref. 0= no) | 0.649*  (-0.091, 1.390) | 0.455  (-0.268, 1.178) | 91 | 58 |
| Eczema 1= yes  (ref. 0= no) | 0.905  (-0.365, 2.175) |  | 147 |  |
| Treatment level 2= TL-2  (ref. 1= TL-1) | 1.330†  (0.138, 2.522) |  | 278 |  |
| Treatment level 3= TL-3  (ref. 1= TL-1) | 1.155†  (0.087, 2.223) |  | 217 |  |
| E-lesion Yes  (ref. No) | 1.066  (-0.192, 2.325) |  | 190 |  |

* p<0.1

† p<0.05

‡ p< 0.01

**Supplement table 3. Regression results for TARC serum and disease burden (cHL patients only)**

|  | **Dependent variable: log(TARC serum)** | | | |
| --- | --- | --- | --- | --- |
|  | Simple regression | Multiple regression | % TARC increase (simple) | % TARC increase (multiple) |
| Age (years) | 0.242‡  (0.141, 0.343) |  | 27 |  |
| Age  >=13 years  (ref. <13 years) | 1.416 ‡  (0.707, 2.125) |  | 312 |  |
| Stage 3= III  (ref. 2= II) | 0.530  (-0.409, 1.468) | 0.648  (-0.236, 1.531) | 70 |  |
| Stage 4= IV  (ref. 2= II) | 0.578  (-0.344, 1.501) | 0.313  (-0.625, 1.251) | 78 | 37 |
| CRP | 0.005  (-0.002, 0.013) |  | 1 |  |
| ESR | 0.010^*^  (0.0003, 0.019) |  | 1 |  |
| ESR 1= yes  (ref. 0= no) | 0.667^*^  (-0.063, 1.398) | 0.218 (-0.646, 1.082) | 95 | 24 |
| Bulky disease 1= yes  (ref. 0= no) | 1.071‡  (0.351, 1.791) | 1.046†  (0.218, 1.875) | 192 | 185 |
| B-symptoms 1= yes  (ref. 0= no) | 0.333  (-0.424, 1.090) | -0.003  (-0.794, 0.789) | 40 | 0 |
| Eczema 1= yes  (ref. 0= no) | 1.213†  (0.110, 2.316) |  | 236 |  |
| Treatment level 2= TL-2  (ref. 1= TL-1) | 0.796  (-0.352, 1.944) |  | 122 |  |
| Treatment level 3= TL-3  (ref. 1= TL-1) | 0.702  (-0.312, 1.716) |  | 102 |  |
| E-lesion Yes  (ref. No) | 0.894  (-0.692, 2.480) |  | 144 |  |

* p<0.1

† p<0.05

‡ p< 0.01
